# Superconductivity in the High‐Entropy Ceramics Ti_0.2_Zr_0.2_Nb_0.2_Mo_0.2_Ta_0.2_C*_x_* with Possible Nontrivial Band Topology

**DOI:** 10.1002/advs.202305054

**Published:** 2023-12-05

**Authors:** Lingyong Zeng, Xunwu Hu, Yazhou Zhou, Mebrouka Boubeche, Ruixin Guo, Yang Liu, Si‐Chun Luo, Shu Guo, Kuan Li, Peifeng Yu, Chao Zhang, Wei‐Ming Guo, Liling Sun, Dao‐Xin Yao, Huixia Luo

**Affiliations:** ^1^ School of Materials Science and Engineering State Key Laboratory of Optoelectronic Materials and Technologies Guangdong Provincial Key Laboratory of Magnetoelectric Physics and Devices Sun Yat‐Sen University No. 135, Xingang Xi Road Guangzhou 510275 China; ^2^ Guangdong Provincial Key Laboratory of Magnetoelectric Physics and Devices Center for Neutron Science and Technology School of Physics Sun Yat‐Sen University Guangzhou 510275 China; ^3^ Institute of Physics Chinese Academy of Sciences Beijing 100190 China; ^4^ Songshan Lake Materials Laboratory University Innovation Town Building A1, Dongguan Guang Dong 523808 China; ^5^ Shenzhen Institute for Quantum Science and Engineering Southern University of Science and Technology Shenzhen 518055 China; ^6^ International Quantum Academy Shenzhen 518048 China; ^7^ School of Electromechanical Engineering Guangdong University of Technology Guangzhou 510006 China

**Keywords:** high‐entropy ceramics, high‐pressure, superconductivity, topological superconductors

## Abstract

Topological superconductors have drawn significant interest from the scientific community due to the accompanying Majorana fermions. Here, the discovery of electronic structure and superconductivity (SC) in high‐entropy ceramics Ti_0.2_Zr_0.2_Nb_0.2_Mo_0.2_Ta_0.2_C*_x_* (*x* = 1 and 0.8) combined with experiments and first‐principles calculations is reported. The Ti_0.2_Zr_0.2_Nb_0.2_Mo_0.2_Ta_0.2_C*_x_* high‐entropy ceramics show bulk type‐II SC with *T*_c_ ≈ 4.00 K (*x* = 1) and 2.65 K (*x* = 0.8), respectively. The specific heat jump (∆*C*/*γT*_c_) is equal to 1.45 (*x* = 1) and 1.52 (*x* = 0.8), close to the expected value of 1.43 for the BCS superconductor in the weak coupling limit. The high‐pressure resistance measurements show a robust SC against high physical pressure in Ti_0.2_Zr_0.2_Nb_0.2_Mo_0.2_Ta_0.2_C, with a slight *T*_c_ variation of 0.3 K within 82.5 GPa. Furthermore, the first‐principles calculations indicate that the Dirac‐like point exists in the electronic band structures of Ti_0.2_Zr_0.2_Nb_0.2_Mo_0.2_Ta_0.2_C, which is potentially a topological superconductor. The Dirac‐like point is mainly contributed by the *d* orbitals of transition metals M and the *p* orbitals of C. The high‐entropy ceramics provide an excellent platform for the fabrication of novel quantum devices, and the study may spark significant future physics investigations in this intriguing material.

## Graphical abstract

The discovery of superconductivity (SC) and topological properties in the Ti_0.2_Zr_0.2_Nb_0.2_Mo_0.2_Ta_0.2_C*_x_* high‐entropy carbide ceramics suggests they are potential topological superconductors. Experiments show the robustness of superconductivity under pressure in the Ti_0.2_Zr_0.2_Nb_0.2_Mo_0.2_Ta_0.2_C HECC. The research results not only expand the new physical properties of high‐entropy carbide ceramics but also provide a new material platform for studying the coupling between SC and topological physics.

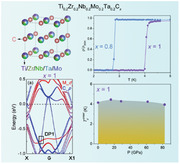

## 1 Introduction

It is always keening for condensed matter scientists to discover new materials and explore their unique physical properties. Superconductivity (SC), in combination with topology, is expected to exhibit new types of quasiparticles, such as non‐Abelian Majorana zero modes or fractional charge and spin currents.^[^ ^1^, ^2^, ^3^, ^4^ ^]^ The experimental realization of topological SC will provide an excellent platform for developing fault‐tolerant quantum computing techniques.^[^ ^5^ ^]^

However, searching for topological superconductors (TSCs) has been challenging. The ways toward realizing topological SC have been adopted: finding SC with nontrivial intrinsic topology or combining the conventional SC with other nontrivial topological band structures (e.g., Bi_2_Se_3_/NbSe_2_, Bi_2_Te_3_/NbSe_2_)^[^ ^6^, ^7^ ^]^ or pressurizing/doping topological materials (topological insulator, topological Weyl semimetal, topological Dirac semimetal).^[^ ^8^, ^9^, ^10^, ^11^, ^12^, ^13^, ^14^, ^15^, ^16^ ^]^ The existence of nontrivial topology in intrinsic superconducting materials offers the possibility to realize TSCs, preventing the complexity of fabricating a proximity‐coupled heterostructure of a superconductor and topological insulator. There have been observations of Majorana zero modes in iron‐based TSCs (FeTe_1‐_ *_x_* Se*_x_*, CaKFe_4_As_4_, and LiFeAs).^[^ ^17^, ^18^, ^19^, ^20^, ^21^, ^22^ ^]^ In addition, both predicted and experimental intrinsic TSCs are exceedingly rare. Most of them can only achieve SC or suitable topological surface states near the Fermi energy (E_F_) by doing. It is highly urgent to search for more intrinsic TSC candidates with high superconducting critical temperature (*T*_c_) and topological surface states near E_F_. In recent, some strong candidates for TSC have been found in compounds formed by the IVA group elements and metals, such as AuSn_4_,^[^ ^23^, ^24^ ^]^ Au_2_Pb,^[^ ^25^ ^]^ PtPb_4_,^[^ ^26^ ^]^ Ta_3_(Sn, Pb),^[^ ^27^ ^]^ BaSn_5_,^[^ ^28^ ^]^ (Ta, Nb)RuSi,^[^ ^29^ ^]^ and binary transition‐metal carbides (TMCs).^[^ ^30^, ^31^, ^32^, ^33^, ^34^ ^]^ Among these TSCs, TMCs have a relatively high *T*_c_. The type‐II Dirac semimetal states were proposed to exist in the band structure of NbC and TaC, which are well‐known comparable high *T_c_* ≈ 11.5 K and 10.6 K superconductors.^[^ ^30^, ^32^, ^34^ ^]^ The first‐principles calculations also indicate that *s*‐wave Bardeen‐Cooper‐Schrieffer (BCS) SC with *T*_c_ ≈ 14 K and nontrivial band topology coexist in cubic *ɑ*‐MoC.^[^ ^33^ ^]^

The high‐entropy alloy (HEA) concept was developed in 2004,^[^ ^35^ ^]^ and since then, an entropy stabilization concept has been used to prepare high‐entropy ceramics (HECs) as well.^[^ ^36^, ^37^ ^]^ HECs are the solid solution of five or more cationic or anionic sublattices with a high configuration entropy.^[^ ^36^, ^37^ ^]^ High‐entropy transition metal carbide ceramics (HECCs), as one of the most intensively researched subsets of these materials, generally exhibit superior mechanical and physical properties, such as high hardness, low thermal conductivity, excellent elevated‐temperature flexural strength, and good resistance to high‐temperature oxidation and wear.^[^ ^36^, ^37^, ^38^, ^39^, ^40^ ^]^ These high‐entropy materials, a form of multi‐carbide solid solution, have drawn widespread attention recently due to their vast potential and broad industrial application prospects. However, the intense research on HECCs has primarily focused on their mechanical properties. The physical properties of HECs, especially SC and topological properties, are still worth exploring.

In this study, HECs of Ti_0.2_Zr_0.2_Nb_0.2_Mo_0.2_Ta_0.2_C*_x_* (*x* = 1 and 0.8) with a single‐phase NaCl‐type structure were prepared by a spark plasma sintering method. We report our discovery and investigation of the HEC superconductors Ti_0.2_Zr_0.2_Nb_0.2_Mo_0.2_Ta_0.2_C*_x_* (*x* = 1 and 0.8), which shows bulk type‐II SC with *T_c_* ≈ 4.00 K (*x* = 1) and 2.65 K (*x* = 0.8), respectively. Considering the extraordinary properties of the HECCs mentioned above, the discovery of SC and topological band structures in these HECCs would make them an excellent platform for novel quantum device fabrication.

## 2 Results and Discussion

**Figure 1:**
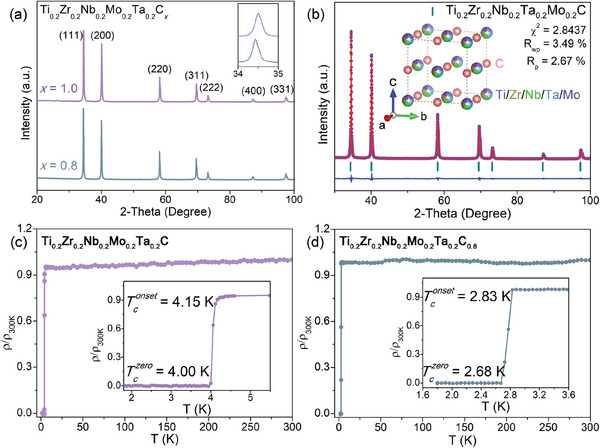
a) PXRD patterns for Ti_0.2_Zr_0.2_Nb_0.2_Mo_0.2_Ta_0.2_C_x_ (*x* = 1 and 0.8) samples. The inset shows the (111) reflections. b) Rietveld refinement profile for Ti_0.2_Zr_0.2_Nb_0.2_Mo_0.2_Ta_0.2_C_*x*_ (*x* = 1 and 0.8) samples. The inset displays the crystal structure. Electrical resistivity as functions of temperature for c) Ti_0.2_Zr_0.2_Nb_0.2_Mo_0.2_Ta_0.2_C and d) Ti_0.2_Zr_0.2_Nb_0.2_Mo_0.2_Ta_0.2_C_0.8_.

**Figure** **1**a exhibits the powder X‐ray diffraction (PXRD) data of the Ti_0.2_Zr_0.2_Nb_0.2_Mo_0.2_Ta_0.2_C*_x_* samples. All the diffraction peaks of Ti_0.2_Zr_0.2_Nb_0.2_Mo_0.2_Ta_0.2_C*_x_* (*x* = 1 and 0.8) are indexed on the space group *Fm* $\bar{3}$ *m*. A decrease in the *x* concentration causes the peak position of (111) to shift toward the lower angle side. Figure 1b shows the Rietveld refinement profile of the Ti_0.2_Zr_0.2_Nb_0.2_Mo_0.2_Ta_0.2_C sample, which displays reliable, refined results with χ^2^ = 2.84, *R*_wp_ = 3.49 %, and *R*_p_ = 2.67 %. The lattice parameters are a = 4.4573(3) Å for *x* = 1 and a = 4.4479(8) Å for *x* = 0.8, respectively. The inset of Figure 1b displays the simplified schematic diagram of the NaCl‐type structure for the Ti_0.2_Zr_0.2_Nb_0.2_Mo_0.2_Ta_0.2_C sample. The carbon element occupies the anion position, while five metal elements likely share a cation position. We further carried out the scanning electron microscopy (SEM) and energy‐dispersive X‐ray spectroscopy (EDX) characterization of Ti_0.2_Zr_0.2_Nb_0.2_Mo_0.2_Ta_0.2_C*_x_* (*x* = 1 and 0.8) fresh cross‐section to check the homogeneity and actual ratio of the compounds. As seen in Figures S1 and S2 (Supporting Information), all constituent elements are homogeneously distributed. The proportion of each metal element is close (See Figure S3, Supporting Information). Note that this method cannot accurately determine its content since carbon has a light mass and is most likely a contaminant in the EDX analysis. Nevertheless, the EDX results showed that the carbon content of the *x* = 1 sample is higher than the carbon content of the *x* = 0.8 sample.

Figure 1c,d shows the temperature dependencies of resistivity for Ti_0.2_Zr_0.2_Nb_0.2_Mo_0.2_Ta_0.2_C*_x_* (*x* = 1 and 0.8) samples. A sharp resistivity drop is observed in both cases, indicating the superconducting transition. The zero‐resistivity was achieved at 4.00 K for *x* = 1 and 2.68 K for *x* = 0.8. The normal resistivity decreases only slightly with a near temperature independent, similar to that observed in HEA superconductors.^[^ ^41^, ^42^ ^]^ The residual resistivity ratio (RRR) value for Ti_0.2_Zr_0.2_Nb_0.2_Mo_0.2_Ta_0.2_C*_x_* (*x* = 1 and 0.8) samples is close to one.

**Figure 2:**
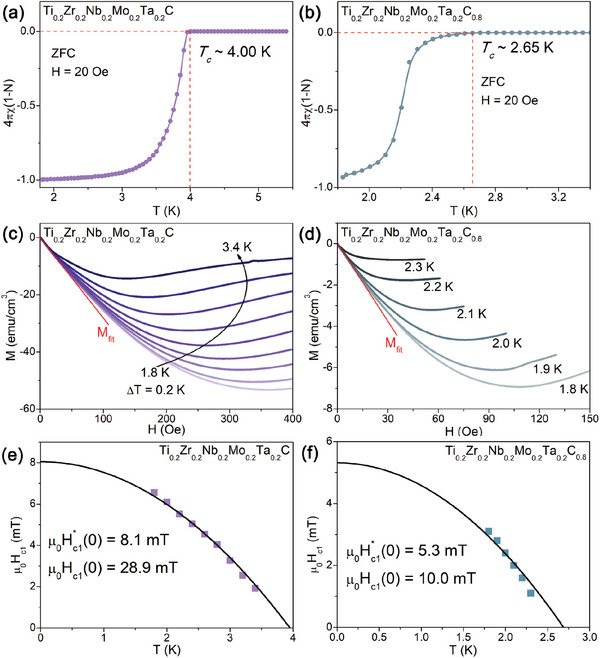
Temperature dependences of the zero‐field‐cooled (ZFC) volume magnetic susceptibility measured in a magnetic field of 20 Oe for a) Ti_0.2_Zr_0.2_Nb_0.2_Mo_0.2_Ta_0.2_C and b) Ti_0.2_Zr_0.2_Nb_0.2_Mo_0.2_Ta_0.2_C_0.8_. The field‐dependent magnetization curves for c) Ti_0.2_Zr_0.2_Nb_0.2_Mo_0.2_Ta_0.2_C and d) Ti_0.2_Zr_0.2_Nb_0.2_Mo_0.2_Ta_0.2_C_0.8_. The temperature‐dependent lower critical fields for e) Ti_0.2_Zr_0.2_Nb_0.2_Mo_0.2_Ta_0.2_C and f) Ti_0.2_Zr_0.2_Nb_0.2_Mo_0.2_Ta_0.2_C_0.8_.

The temperature‐dependent magnetic susceptibility was measured under 20 Oe in the zero‐filed cooling (ZFC) for *x* = 1 (**Figure** **2**a) and *x* = 0.8 (Figure 2b). To get a more accurate value of the superconducting shielding fraction, the demagnetization factors (N) for Ti_0.2_Zr_0.2_Nb_0.2_Mo_0.2_Ta_0.2_C*_x_* samples are estimated to be 0.72 (*x* = 1) and 0.47 (*x* = 0.8) respectively, by using N = 1 + 1/(4πs), where s is the slope of linear fitting in the field‐dependent volume magnetization curve at 1.8 K. We also calculate the theoretical N value using the equation $N^{-1}=1+\frac{3}{4}\frac{c}{a}\left(1+\frac{a}{b}\right)$,^[^ ^43^ ^]^ where 2a × 2b × 2c is the geometric parameters of the cuboid sample. The theoretical N values are calculated to be 0.67 (*x* = 1) and 0.43 (*x* = 0.8), respectively, consistent with the actual values. The resulting diamagnetic signal with a clear transition to a superconducting state is close to 100% Meissner volume fraction, indicating the bulk nature of SC in Ti_0.2_Zr_0.2_Nb_0.2_Mo_0.2_Ta_0.2_C*_x_* samples. The onset diamagnetic transition temperatures are 4.00 K for *x* = 1 and 2.65 K for *x* = 0.8, which agree well with that from resistivity data.

Figure 2c,d shows isothermal magnetization curves over different temperatures below the *T_c_* for Ti_0.2_Zr_0.2_Nb_0.2_Mo_0.2_Ta_0.2_C*_x_* (*x* = 1 and 0.8) samples. The lower critical fields (μ_0_H_c1_(0)) are obtained from the fields where the M(H) deviates from the linearly field‐dependent behavior (Meissner line), i.e., the magnetic flux starts to penetrate the SC body. All the uncorrected lower critical fields, μ_0_H_c1_*, with the corresponding temperatures, are plotted in Figure 2e for *x* = 1 and Figure 2f for *x* = 0.8. The data points are modeled with the GL relation: $\mu_{0}H_{c1}^{*}\left(T\right)=\mu_{0}H_{c1}^{*}\left(0\right)/\left(1-{\left(T/T_{c}\right)}^{2}\right)$ giving μ_0_H_c1_*(0) = 8.1(1) mT for *x* = 1 and μ_0_H_c1_*(0)  = 5.3(1) mT for *x* = 0.8. Considering the demagnetization factor, the real μ_0_H_c1_(0) can be deduced from the μ_0_H_c1_* with the formula μ_0_H_c1_(0) = μ_0_H_c1_*(0)/(1‐N). The estimated μ_0_H_c1_(0) = 28.9(6) mT for *x* = 1 and μ_0_H_c1_(0) = 10.0(2) mT for *x* = 0.8.

**Figure 3:**
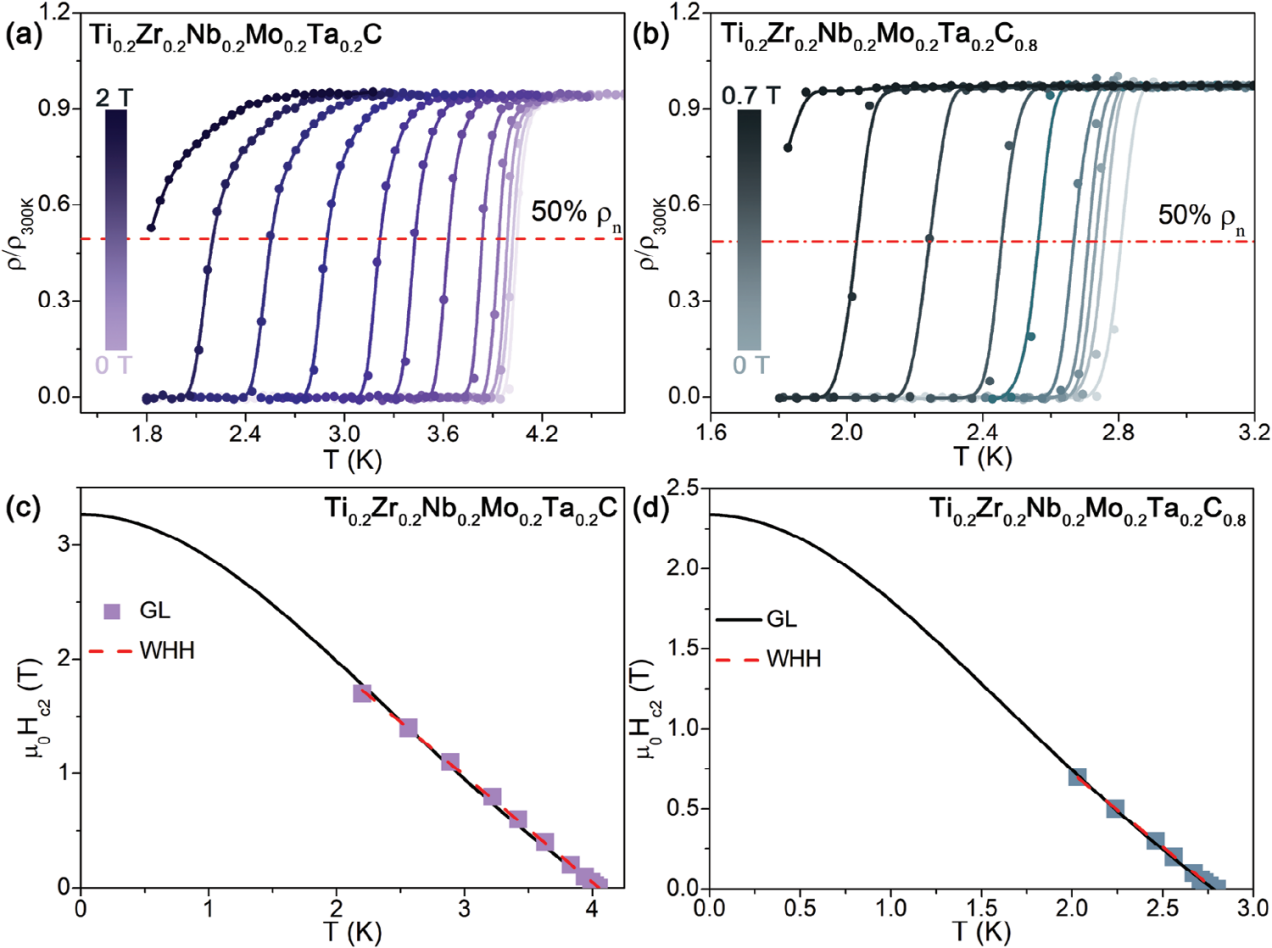
Low‐temperature resistivity measurements in a variety of magnetic fields for a) Ti_0.2_Zr_0.2_Nb_0.2_Mo_0.2_Ta_0.2_C and b) Ti_0.2_Zr_0.2_Nb_0.2_Mo_0.2_Ta_0.2_C_0.8_. The temperature‐dependent upper critical field for c) Ti_0.2_Zr_0.2_Nb_0.2_Mo_0.2_Ta_0.2_C and d) Ti_0.2_Zr_0.2_Nb_0.2_Mo_0.2_Ta_0.2_C_0.8_.

The low‐temperature resistivity under different magnetic fields for Ti_0.2_Zr_0.2_Nb_0.2_Mo_0.2_Ta_0.2_C*_x_* (*x* = 1 and 0.8) samples is presented in **Figure** **3**a,b, respectively. Upon applying the magnetic field, the *T*_c_ decreases steadily for both HECs. Figure 3c,d shows the upper critical fields μ_0_H_c2_ plotted as a function of the estimated *T_c_* values for Ti_0.2_Zr_0.2_Nb_0.2_Mo_0.2_Ta_0.2_C*_x_* (*x* = 1 and 0.8) samples. The zero temperature for a type‐II superconductor in dirty limit can be calculated with the Werthamer Helfand Hohenberg (WHH) theory: μ_0_H_c2_(0) = −0.693*T_c_* ($\frac{d\mu_{0}H_{c2}}{dT}$)|*_T = Tc_*. The extrapolated slopes near *T*_c_ are $\frac{d\mu_{0}H_{c2}}{dT}$ = −0.93(3) T/K for *x* = 1, and $\frac{d\mu_{0}H_{c2}}{dT}$ = −0.92(7) T/K for *x* = 0.8. Thus, based on the slope and *T*_c_, we have μ_0_H_c2_(0) = 2.5(9) T for *x* = 1 and 1.7(0) T for *x* = 0.8. The μ_0_H_c2_(0) is also deduced by extrapolating the data based on the GL model: $\mu_{0}H_{c2}\;\left(T\right)=\mu_{0}\;H_{c2}\left(0\right)*\frac{1-{\left(T/T_{c}\right)}^{2}}{1+{\left(T/T_{c}\right)}^{2}}$, giving 3.2(6) T and 2.3(4) T for *x* = 1 and *x* = 0.8, respectively. According to the equation μ_0_H^P^ = 1.85*T_c_, the Pauli paramagnetic limits are 7.40 T and 4.90 T for *x* = 1 and *x* = 0.8, respectively.

Various superconducting parameters can be calculated using μ_0_H_c1_(0) and μ_0_H_c2_(0). First, according to the formula $\xi_{\mathrm{GL}}^{2}\left(0\right)=\frac{\Phi_{0}}{2\pi \mu_{0}H_{c2}\left(0\right)}$, where Ф_0_ = h/2e represents the flux quantum, the GL coherence length (ξ_*GL*_(0)) is determined to be 100.5(3) Å and 118.6(5) Å for *x* 1 and *x* = 0.8, respectively. Second, the GL penetration depth at zero K, *λ_GL_* (0), is obtained 1186 Å and 2192 Å for *x* = 1 and *x* = 0.8, respectively, using the expression: $\mu_{0}H_{c1}\;\left(0\right)=\frac{\Phi_{0}}{4\pi \lambda_{\mathrm{GL}}^{2}\left(0\right)}\;\ln\frac{\lambda_{\mathrm{GL}}\left(0\right)}{\xi_{\mathrm{GL}}\left(0\right)}$. Third, the GL parameter, *K_GL_* (0) = $\frac{\lambda_{GL}\left(0\right)}{\xi_{GL}\left(0\right)}$, can be estimated to be 11.8 (*x* = 1) and 18.5 (*x* = 0.8), which is larger than 1/√2, suggesting that Ti_0.2_Zr_0.2_Nb_0.2_Mo_0.2_Ta_0.2_C*_x_* HECs are strongly type‐II superconductors. Table S1 (Supporting Information) summarizes all the gathered normal and superconducting parameters for Ti_0.2_Zr_0.2_Nb_0.2_Mo_0.2_Ta_0.2_C*_x_* samples. For comparison, the relevant superconducting parameters of previously reported HECCs are also listed in Table S1 (Supporting Information).^[^ ^44^, ^45^ ^]^

**Figure 4:**
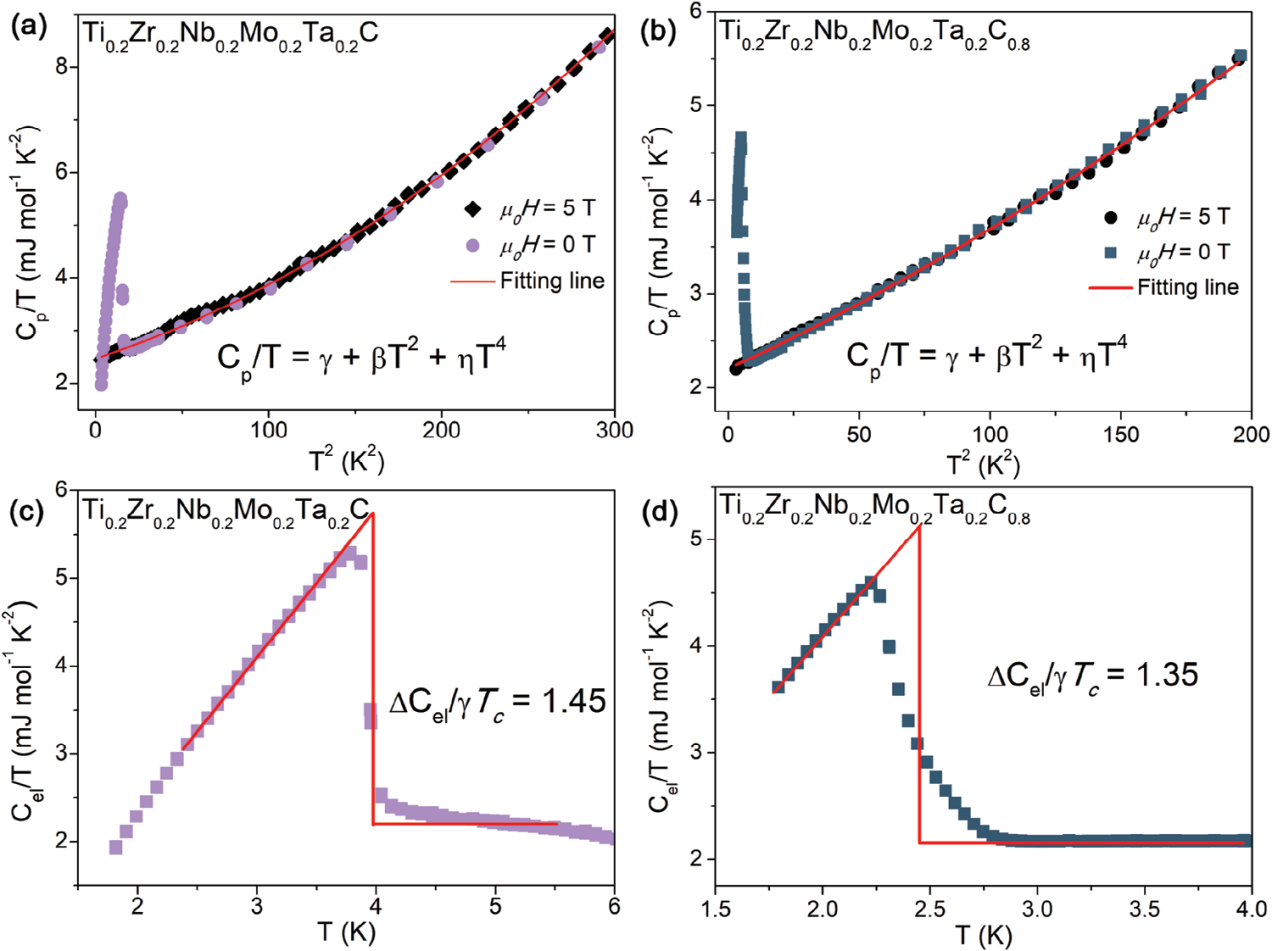
C_p_/T versus T^2^ under 0 T and 5 T for a) Ti_0.2_Zr_0.2_Nb_0.2_Mo_0.2_Ta_0.2_C and b) Ti_0.2_Zr_0.2_Nb_0.2_Mo_0.2_Ta_0.2_C_0.8_. Temperature‐dependent normalized electronic specific heats for c) Ti_0.2_Zr_0.2_Nb_0.2_Mo_0.2_Ta_0.2_C and d) Ti_0.2_Zr_0.2_Nb_0.2_Mo_0.2_Ta_0.2_C_0.8_.

The low‐temperature specific heat measurements under applied magnetic fields of 0 and 5 T were performed to confirm the bulk nature of the SC. The obvious anomaly in the 0 T heat capacity (**Figure** **4**a,b), corresponding with the emergence of the superconducting state, can be observed in Ti_0.2_Zr_0.2_Nb_0.2_Mo_0.2_Ta_0.2_C*_x_* (*x* = 1 and 0.8) samples. Based on the equal entropy construction, we find that the *T*_c_ = 3.98 K for *x* = 1 sample and *T*_c_ = 2.49 K for *x* = 0.8 sample. The heat capacity data is fitted well with the Debye model, C_p_/T = *γ* + *β*T^2^ + *η*T^4^, where two‐term, *β*T^2^ + ηT^4^, are used to express the phonon contribution, and *γ* is the normal state electronic specific heat coefficient. The best fits give *γ* = 2.471(5) mJ mol^−1^ K^−2^, *β* = 0.010(6) mJ mol^−1^ K^−4^ for *x* = 1 sample, and *γ* = 2.206(2) mJ mol^−1^ K^−2^, *β* = 0.012(9) mJ mol^−1^ K^−4^ for *x* = 0.8 sample. Then, another important superconducting parameter, specific heat jump (∆*C*/*γT*_c_) at *T*_c_, can be determined. The ∆*C*/*γT*_c_ is equal to 1.45 (*x* = 1) and 1.35 (*x* = 0.8), close to the expected value of 1.43 for the BCS superconductor, verifying the bulk nature of the SC in these HECs.

Then we estimate the Debye temperature (Θ_D_) through the equation Θ_D_ = (12π^4^ *nR*/5*β*)^1/3^, where R has a value of 8.31 J mol^−1^ K as the gas constant and n is the number of atoms per formula unit (n = 1 + *x* for Ti_0.2_Zr_0.2_Nb_0.2_Mo_0.2_Ta_0.2_C*_x_* samples). It yields Θ_D_ = 715 K and 647 K for *x* = 1 and 0.8, respectively. With the *T*_c_ and Θ_D_, we can obtain the electron‐phonon coupling constant (*λ_ep_*) through the McMillan formula, $\lambda_{ep}=\frac{1.04+\mu^{*}\ln\left(\frac{\Theta_{D}}{1.45T_{c}}\right)}{\left(1-0.62\mu^{*}\right)\ln\left(\frac{\Theta_{D}}{1.45T_{c}}\right)-1.04}$ where μ* represents the Coulomb pseudopotential parameter and is typically given a value of 0.13.^[^ ^46^, ^47^ ^]^ Based on the obtained values, the superconducting parameter *λ_ep_* = 0.49 for *x* = 1 and *λ*_ep_ = 0.46 for *x* = 0.8. The *λ*_ep_ values suggest that Ti_0.2_Zr_0.2_Nb_0.2_Mo_0.2_Ta_0.2_C*_x_* HECs are weak‐coupling superconductors. In crystalline materials, electron–phonon coupling is a ubiquitous many‐body interaction that drives conventional SC. The phonon mechanism is responsible for the electron–electron coupling and, hence, the cause of SC.^[^ ^48^, ^49^, ^50^ ^]^ In the Ti_0.2_Zr_0.2_Nb_0.2_Mo_0.2_Ta_0.2_C*_x_* system, the electron–phonon coupling strength weakens as the *T*_c_ decreases.

**Figure 5:**
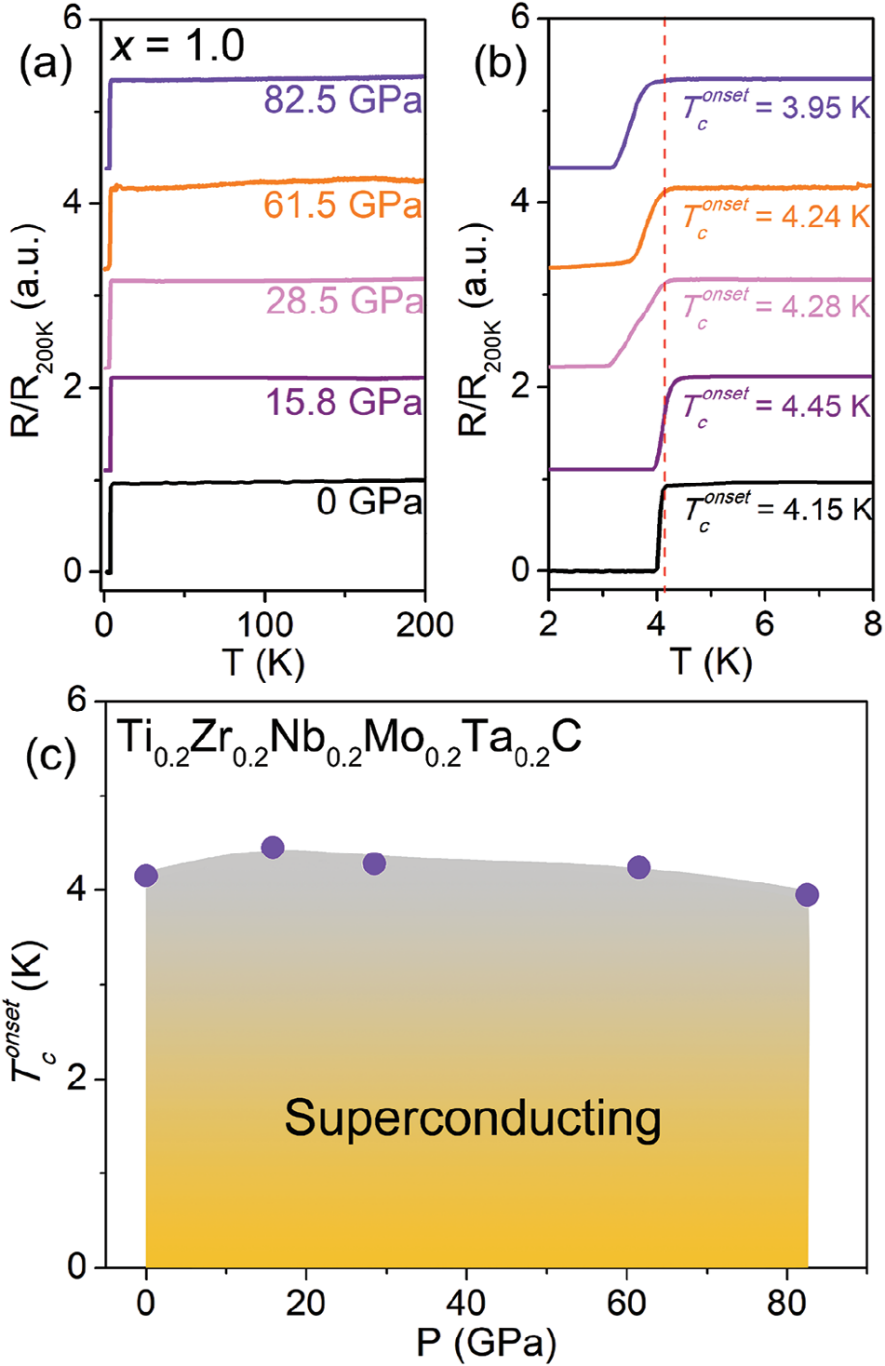
a) Temperature dependence of resistance in Ti_0.2_Zr_0.2_Nb_0.2_Mo_0.2_Ta_0.2_C HEC in the pressure range of 0–82.5 GPa. b) Temperature dependence of resistance in Ti_0.2_Zr_0.2_Nb_0.2_Mo_0.2_Ta_0.2_C HEC near the superconducting transition. c) The phase diagram in Ti_0.2_Zr_0.2_Nb_0.2_Mo_0.2_Ta_0.2_C HEC is a function of pressure and temperature.

To further investigate the SC of HEC, we performed the high‐pressure resistance measurements for Ti_0.2_Zr_0.2_Nb_0.2_Mo_0.2_Ta_0.2_C HEC. **Figure** **5**a shows the typical resistance curves of Ti_0.2_Zr_0.2_Nb_0.2_Mo_0.2_Ta_0.2_C HEC under various pressures up to 82.5 GPa. It is seen that the superconducting transitions of Ti_0.2_Zr_0.2_Nb_0.2_Mo_0.2_Ta_0.2_C HEC subjected to different pressures are sharp, and the zero‐resistance state remains present throughout the full range of pressures applied (see Figure 5b). The *T*_c_ shows only a slight change from its ambient‐pressure value of 4.15 K to 3.95 K at 82.5 GPa. The pressure‐dependent *T*_c_ for Ti_0.2_Zr_0.2_Nb_0.2_Mo_0.2_Ta_0.2_C HEC is mapped in the phase diagram in Figure 5c. We see a robust SC against high physical pressure in Ti_0.2_Zr_0.2_Nb_0.2_Mo_0.2_Ta_0.2_C HEC, with a slight *T*_c_ variation of 0.3 K within 82.5 GPa. A similar phenomenon was also observed in (TaNb)_0.67_(HfZrTi)_0.33_ HEA.^[^ ^51^ ^]^ This makes superconducting HECs also promising candidates for new applications under extreme conditions.

**Figure 6:**
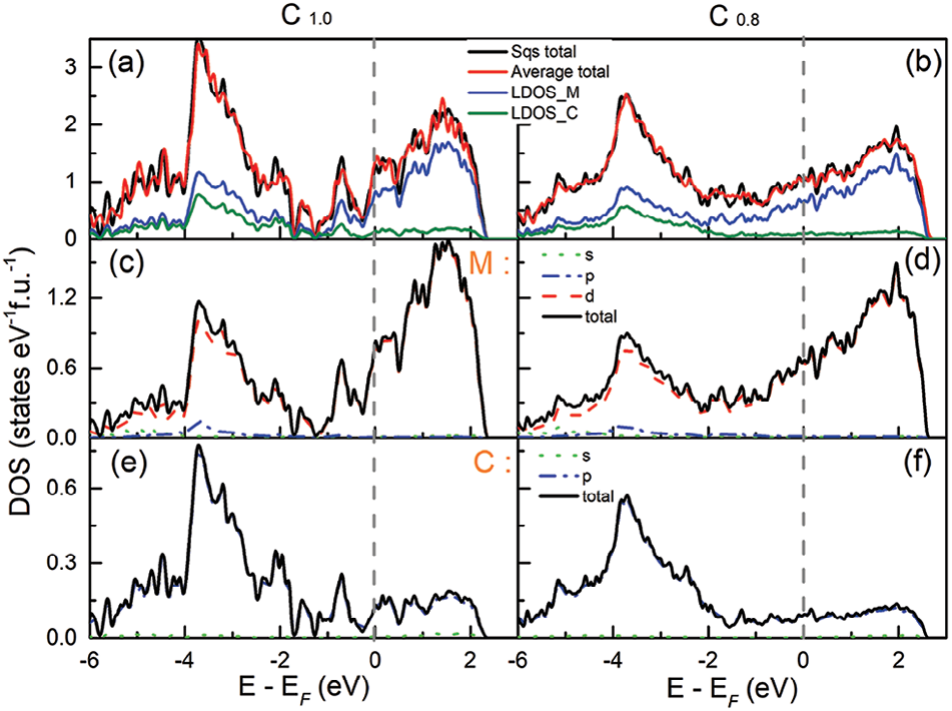
The Sqs and average DOS of Ti_0.2_Zr_0.2_Nb_0.2_Mo_0.2_Ta_0.2_C*_x_* calculated by considering four assumed structures built by “mcsqs” code (*x* = 1 for a), c), and e), *x* = 0.8 for b), d), and f)). a,b) Total and local DOS of Ti_0.2_Zr_0.2_Nb_0.2_Mo_0.2_Ta_0.2_C*_x_*. c–f) Projected DOS with angular momentum decomposition of each element. The gray dashed lines indicate the Fermi level.

The lattice parameter of the Ti_0.2_Zr_0.2_Nb_0.2_Mo_0.2_Ta_0.2_C fitted by the Birch‐Murnaghan equation of state is 4.484 Å, which is inconsistent with the experimental lattice parameter (a = 4.4573(3) Å) (see Figure S4, Supporting Information). For simplicity, the lattice parameters of Ti_0.2_Zr_0.2_Nb_0.2_Mo_0.2_Ta_0.2_C*_x_* are fixed to the experimentally refined lattice constants. The total density of states (TDOS), the local density of states (DOS), and the partial DOS for Ti_0.2_Zr_0.2_Nb_0.2_Mo_0.2_Ta_0.2_C*_x_* are shown in **Figure** **6**. In this work, the supercell contains 64 atoms, so the experimental doping ratio r_Ti_ = r_Zr_ = r_Nb_ = r_Mo_ = r_Ta_ = 0.2 cannot be obtained. We consider four different atomic arrangements’ structural configurations (the doping ratio is equal to 0.1875 for three elements and 0.21875 for the remaining two.) for investigating the influence of the disorder on the electronic properties of the Ti_0.2_Zr_0.2_Nb_0.2_Mo_0.2_Ta_0.2_C*_x_*. The overall shape of the averaged TDOS for *x* = 1 and *x* = 0.8 are pretty similar, while is quite different near the Fermi level. The TDOS passing through the Fermi level suggests its typical metallic properties (see Figure 6a,b). The local DOS diagram shows that the Ti, Zr, Nb, Mo, and Ta atoms are the most significant contributors to TDOS near the Fermi level. In contrast, the contribution from the C atoms is relatively modest. The d orbital of M and p orbital of C electrons are highly hybridized below the Fermi level. As displayed in Figure 6c–f, the projected DOS with angular momentum reveals that the *d*‐electrons of M elements are the main contributions, i.e., 3*d* for Ti, 4*d* for Zr, Nb, Mo, and 5*d* for Ta. These results indicate that the SC may mainly originate from the *d*‐electrons of Ti, Zr, Nb, Mo, and Ta.

**Figure 7:**
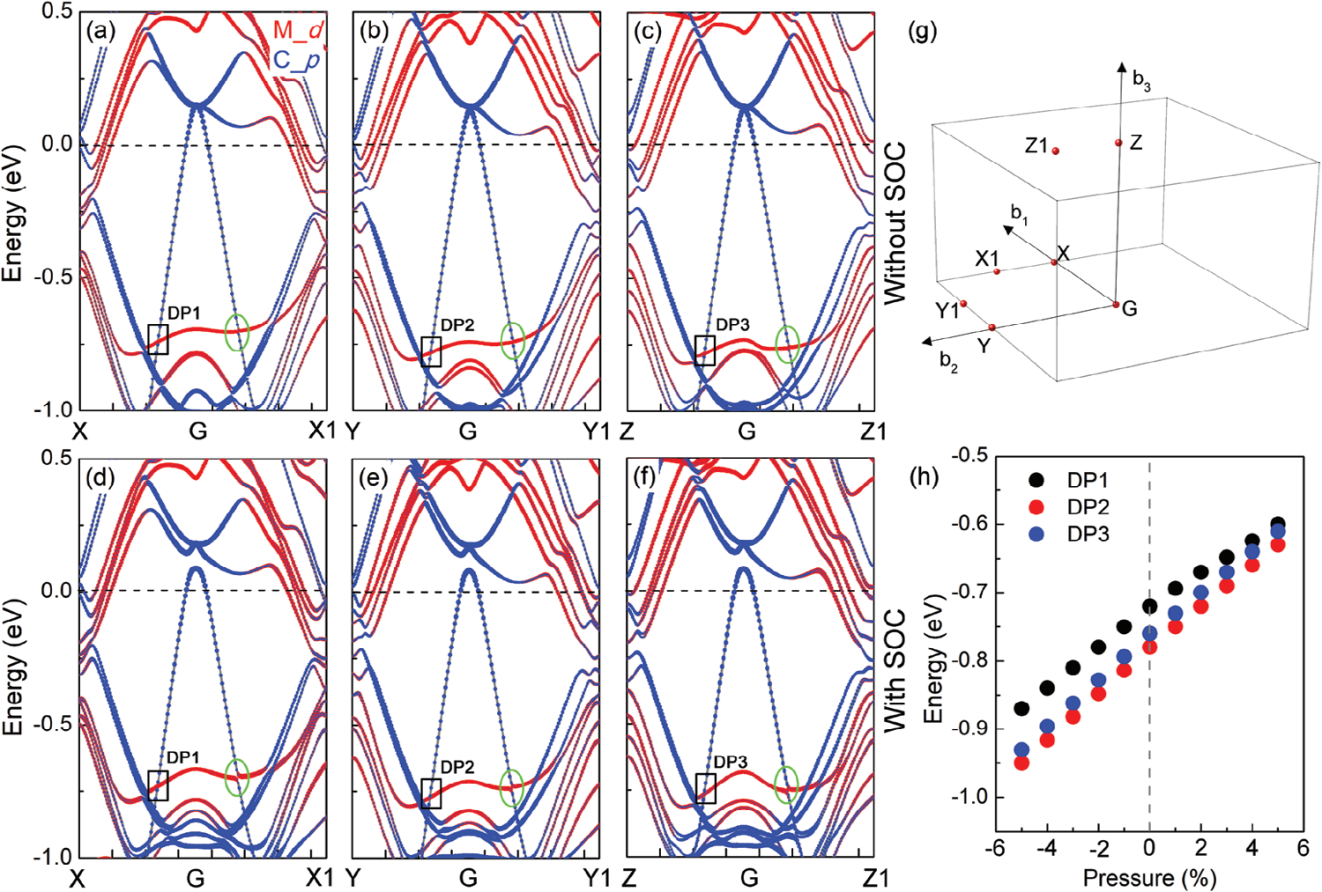
Electronic band structures of Ti_0.2_Zr_0.2_Nb_0.2_Mo_0.2_Ta_0.2_C (*x* = 1) calculated by a–c) ignoring and by d–f) considering the spin‐orbit coupling (SOC). The black rectangles indicate the type‐II DPs. The three representative crystal structures are used in (a–c), shown in Figure S4. g) The first Brillouin zone of Ti_0.2_Zr_0.2_Nb_0.2_Mo_0.2_Ta_0.2_C. h) Strain dependence of the relative energy at the position of type‐II DPs. The Fermi level is indicated by the gray dashed lines. An amplified scaling factor of five is used for the C element in (a–f).

**Figure** **7** shows the electronic band structures of Ti_0.2_Zr_0.2_Nb_0.2_Mo_0.2_Ta_0.2_C (*x* = 1). We first study the band structures of Ti_0.2_Zr_0.2_Nb_0.2_Mo_0.2_Ta_0.2_C without SOC. The three different structures are displayed in Figure S5 (Supporting Information). As is shown in Figure 7a–c, there exist six linear band intersections along G–X, G–Y, and G–Z directions at ≈–0.75 eV (Type ‐II Dirac‐like points (DPs) are denoted by the black circle rectangles). As shown in Figure 7d,e, the linear band intersection along G–X, G–Y, and G–Z directions is not split by considering the SOC, while three linear band intersections along the G–X1, G–Y1, and G–Z1 directions are lightly split (denoted by green circles). The projected band structures of Ti_0.2_Zr_0.2_Nb_0.2_Mo_0.2_Ta_0.2_C show that the DPs mainly contribute from the *d* orbitals of transition metals M and the *p* orbitals of C. As shown in Figure 7h, the positions of the DPs are sensitive to the strain. Therefore, we propose that the Ti_0.2_Zr_0.2_Nb_0.2_Mo_0.2_Ta_0.2_C is a topological superconductor candidate. Compared with the case of Ti_0.2_Zr_0.2_Nb_0.2_Mo_0.2_Ta_0.2_C, the trivial electronic band structures of Ti_0.2_Zr_0.2_Nb_0.2_Mo_0.2_Ta_0.2_C_0.8_ are shown in Figure S6 (Supporting Information).

## 3 Conclusion

In conclusion, we have reported synthesized and characterized new HEC superconductors Ti_0.2_Zr_0.2_Nb_0.2_Mo_0.2_Ta_0.2_C*_x_*. Both Ti_0.2_Zr_0.2_Nb_0.2_Mo_0.2_Ta_0.2_C (*x* = 1) and Ti_0.2_Zr_0.2_Nb_0.2_Mo_0.2_Ta_0.2_C_0.8_ (*x* = 0.8) are discovered to be bulk superconductors with *T*_c_ values of 4.00 and 2.65 K, respectively. The derived superconducting parameters show that Ti_0.2_Zr_0.2_Nb_0.2_Mo_0.2_Ta_0.2_C*_x_* are type‐II BCS weak‐coupling superconductors. We observed a robust SC against high physical pressure in Ti_0.2_Zr_0.2_Nb_0.2_Mo_0.2_Ta_0.2_C HEC, with a slight *T*_c_ variation of 0.3 K within 82.5 GPa. The first‐principles calculations show that the DPs exist in the electronic band structures of Ti_0.2_Zr_0.2_Nb_0.2_Mo_0.2_Ta_0.2_C. The DPs are mainly contributed by the *p* orbitals of C and the *d* orbitals of transition metals M. The research results not only expand the new physical properties of HECCs but also provide a new material platform for studying the coupling between SC and topological physics.

## 4 Experimental Section

The Ti_0.2_Zr_0.2_Nb_0.2_Ta_0.2_Mo_0.2_C*_x_* starting powders were synthesized via the carbothermal reduction method,^[^ ^52^ ^]^ utilizing the molar ratio of MoO_3_, Ta_2_O_5_, Nb_2_O_5_, ZrO_2_, TiO_2_, and graphite powders as the precursor materials. These molar ratios used were based on the equation as follows: TiO_2_ + ZrO_2_ + 0.5Nb_2_O_5_ + 0.5Ta_2_O_5_ + MoO_3_ + (5*x* + 12)C → 5 Ti_0.2_Zr_0.2_Nb_0.2_Ta_0.2_Mo_0.2_C*_x_* + 12CO (g) (where *x* = 1 or 0.8). The raw powders were subjected to ball milling in anhydrous ethanol for a duration of 24 h, employing Si_3_N_4_ balls. Subsequently, the powder mixtures were dried using a rotary evaporator and sieved through a 100‐mesh sieve. The resulting precursors then were placed in a graphite vacuum furnace and subjected to a temperature of 1650 °C for 3 h to synthesize Ti_0.2_Zr_0.2_Nb_0.2_Ta_0.2_Mo_0.2_C*_x_* powders. A suitable quantity of these powders was then loaded into a graphite mold within the spark plasma sintering (SPS) furnace to sinter Ti_0.2_Zr_0.2_Nb_0.2_Ta_0.2_Mo_0.2_C*_x_* samples. The powders were then heated to a temperature of 2000 °C for a duration of 10 min, with a heating and cooling rate of 100 °C min^−1^, under an atmosphere of one atm Ar.

PXRD data were taken on the MiniFlex of Rigaku at a scanning rate of 1^o^min^−1^. Through Rietveld refinements in Fullprof suit software, lattice parameters were obtained. Chemical composition was estimated with SEM‐EDX with an electron acceleration voltage of 20 KV. The temperature‐dependent electrical resistivity magnetic susceptibility and heat capacity were measured by a physical property measurement system (PPMS, Quantum Design. Inc.). The resistance measurements were performed with a four‐probe method. The magnetization and heat capacity measurements use small pieces of sample. The high‐pressure resistance measurements were performed at the high‐pressure station equipped with a diamond anvil cell at the Synergetic Extreme Condition User Facility. In the measurements, the standard four‐probe electrodes (platinum foils) were applied to the samples, and the pressure was determined by the ruby fluorescence method.^[^ ^53^ ^]^ For all resistivity measurements at ambient pressure, platinum wires were connected to the sample with silver paint.

We performed the calculations using the experimental lattice structure parameters. The Ti/Zr/Nb/Mo/Ta and C atoms were fixed in the observed positions 4a (0, 0, 0) and 4b (1/2, 1/2, 1/2), respectively. The chemically disordered solutions of Ti_0.2_Zr_0.2_Nb_0.2_Hf_0.2_Ta_0.2_C*_x_* HECs were modeled by the “mcsqs” code of the Alloy Theoretic Automated Toolkit (ATAT).^[^ ^54^ ^]^ The 2 × 2 × 2 supercell with 64 atoms was adopted. The electronic structure properties calculations are performed using the Vienna *ab “initio”* simulation package (VASP) code^[^ ^55^, ^56^ ^]^ based on density functional theory (DFT). For exchange‐correlation functions, the generalized gradient approximation (GGA) in the form of Perdew‐Burke‐Ernzerhof (PBE)^[^ ^57^ ^]^ is adopted. The projector augmented‐wave (PAW) method^[^ ^58^ ^]^ with a 400 eV plane‐wave cutoff energy is employed. For Brillouin zone sampling, a Γ‐centered 5 × 5 × 5 k‐points mesh within the Monkhorst‐Pack scheme was used in the self‐consistent process. Convergence criteria for the electronic self‐consistent iteration are set to 10^−6^ eV. Spin–orbit coupling (SOC) is used in the calculations of electronic band structure properties.

## Conflict of Interest

The authors declare no conflict of interest.

## Supporting information

Supporting Information

## Data Availability

The data that support the findings of this study are available on request from the corresponding author. The data are not publicly available due to privacy or ethical restrictions.

## References

[1] N. B. Kopnin , M. M. Salomaa , Phys. Rev. B 1991, 44, 9667. 10.1103/physrevb.44.9667 9998953

[2] N. Read , D. Green , Phys. Rev. B 2000, 61, 10267.

[3] J. R. Badger , Y. Quan , M. C. Staab , S. Sumita , A. Rossi , K. P. Devlin , K. Neubauer , D. S. Shulman , J. C. Fettinger , P. Klavins , S. M. Kauzlarich , D. Aoki , I. M. Vishik , W. E. Pickett , V. Taufour , Commun. Phys. 2022, 5, 22.

[4] X.‐L. Qi , S.‐C. Zhang , Rev. Mod. Phys. 2011, 83, 1057.

[5] A. Y. Kitaev , Ann. Phys. 2003, 303, 2.

[6] J.‐P. Xu , M.‐X. Wang , Z. L. Liu , J.‐F. Ge , X. Yang , C. Liu , Z. A Xu , D. Guan , C. L. Gao , D. Qian , Y. Liu , Q.‐H. Wang , F.‐C Zhang , Q.‐K. Xue , J.‐F. Jia , Phys. Rev. Lett. 2015, 114, 017001. 25615497 10.1103/PhysRevLett.114.017001

[7] M.‐X. Wang , C. Liu , J.‐P. Xu , F. Yang , L. Miao , M.‐Y. Yao , C. L. Gao , C. Shen , X. Ma , X. Chen , Z.‐A. Xu , Y. Liu , S.‐C. Zhang , D. Qian , J.‐F. Jia , Q.‐K. Xue , Science 2012, 336, 52. 22422860 10.1126/science.1216466

[8] Y. S. Hor , A. J. Williams , J. G. Checkelsky , P. Roushan , J. Seo , Q. Xu , H. W. Zandbergen , A. Yazdani , N. P. Ong , R. J. Cava , Phys. Rev. Lett. 2010, 104, 057001. 20366785 10.1103/PhysRevLett.104.057001

[9] P. P. Kong , J. L. Zhang , S. J. Zhang , J. Zhu , Q. Q. Liu , R. C. Yu , Z. Fang , C. Q. Jin , W. G. Yang , X. H. Yu , J. L. Zhu , Y. S. Zhao , J. Phys. Condens. Matter. 2013, 25, 362204. 23945091 10.1088/0953-8984/25/36/362204

[10] X. Zhang , K.‐H. Jin , J. Mao , M. Zhao , Z. Liu , F. Liu , npj Comput Mater. 2021, 7, 44.

[11] L. A Wray , S.‐Y. Xu , Y. Xia , Y. S. Hor , D. Qian , A. V. Fedorov , H. Lin , A. Bansil , R. J. Cava , M. Z Hasan , Nat. Phys. 2010, 6, 855.

[12] G. Du , J. Shao , X. Yang , Z. Du , D. Fang , J. Wang , K. Ran , J. Wen , C. Zhang , H. Yang , Y. Zhang , H.‐H. Wen , Nat. Commun. 2017, 8, 14466. 28198378 10.1038/ncomms14466 PMC5316857

[13] Z. Liu , X. Yao , J. Shao , M. Zuo , L. Pi , S. Tan , C. Zhang , Y. Zhang , J. Am. Chem. Soc. 2015, 137, 10512. 26262431 10.1021/jacs.5b06815

[14] S. Sasaki , Z. Ren , A. A. Taskin , K. Segawa , L. Fu , Y. Ando , Phys. Rev. Lett. 2012, 109, 217004. 23215610 10.1103/PhysRevLett.109.217004

[15] P. Hosur , X. Dai , Z. Fang , X.‐L. Qi , Phys. Rev. B 2014, 90, 045130.

[16] Z. Chi , X. Chen , C. An , L. Yang , J. Zhao , Z. Feng , Y. Zhou , Y. Zhou , C. Gu , B. Zhang , Y. Yuan , C. Kenney‐Benson , W. Yang , G. Wu , X. Wan , Y. Shi , X. Yang , Z. Yang , npj Quantum Mater. 2018, 3, 28.

[17] S. Zhu , L. Kong , L. Cao , H. Chen , M. Papaj , S. Du , Y. Xing , W. Liu , D. Wang , C. Shen , F. Yang , J. Schneeloch , R. Zhong , G. Gu , L. Fu , Y.‐Y. Zhang , H. Ding , H.‐J. Gao , Science 2020, 367, 189. 31831637 10.1126/science.aax0274

[18] C.‐K. Chiu , T. Machida , Y. Huang , T. Hanaguri , F.‐C. Zhang , Sci. Adv. 2020, 6, eaay0443. 32158938 10.1126/sciadv.aay0443 PMC7048414

[19] G. Xu , B. Lian , P. Tang , X.‐L. Qi , S.‐C. Zhang , Phys. Rev. Lett. 2016, 117, 047001. 27494494 10.1103/PhysRevLett.117.047001

[20] W. Liu , L. Cao , S. Zhu , L. Kong , G. Wang , M. Papaj , P. Zhang , Y.‐B. Liu , H. Chen , G. Li , F. Yang , T. Kondo , S. Du , G.‐H. Cao , S. Shin , L. Fu , Z. Yin , H.‐J. Gao , H. Ding , Nat. Commun. 2020, 11, 5688. 33173056 10.1038/s41467-020-19487-1 PMC7655862

[21] L. Kong , L. Cao , S. Zhu , M. Papaj , G. Dai , G. Li , P. Fan , W. Liu , F. Yang , X. Wang , S. Du , C. Jin , L. Fu , H.‐J. Gao , H. Ding , Nat. Commun. 2021, 21, 4146. 10.1038/s41467-021-24372-6 PMC8260634 34230479

[22] M. Li , G. Li , L. Cao , X. Zhou , X. Wang , C. Jin , C.‐K. Chiu , S. J. Pennycook , Z. Wang , H.‐J. Gao , Nature 2022, 606, 890. 35676489 10.1038/s41586-022-04744-8

[23] D. Shen , C. N. Kuo , T. W. Yang , I. N. Chen , C. S. Lue , L. M. Wang , Commun. Mater. 2020, 1, 56.

[24] N. K. Karn , M. M. Sharma , V. P. S. Awana , Supercond. Sci. Tech. 2022, 35, 114002.

[25] F. Martín‐Vega , E. Herrera , B. Wu , V. Barrena , F. Mompeán , M. García‐Hernández , P. C. Canfield , A. M. Black‐Schaffer , J. J. Baldoví , I. Guillamón , H. Suderow , Phys. Rev. Res. 2022, 4, 023241.

[26] C. Q. Xu , B. Li , L. Zhang , J. Pollanen , X. L. Yi , X. Z. Xing , Y. Liu , J. H. Wang , Z. Zhu , Z. X. Shi , X. Xu , X. Ke , Phys. Rev. B 2021, 104, 125127.

[27] M. Kim , C.‐Z. Wang , K.‐M. Ho , Phys. Rev. B 2019, 99, 224506.

[28] L. Han , X. Shi , J. Jiao , Z. Yu , X. Wang , N. Yu , Z. Zou , J. Ma , W. Zhao , W. Xia , Y. Guo , Chinese Phys. Lett. 2022, 39, 067101.

[29] T. Shang , J. Zhao , L.‐H. Hu , J. Ma , D. J. Gawryluk , X. Zhu , H. Zhang , Z. Zhen , B. Yu , Y. Xu , Q. Zhan , E. Pomjakushina , M. Shi , T. Shiroka , Sci. Adv. 2022, 8, eabq6589. 36306356 10.1126/sciadv.abq6589 PMC9616505

[30] T. Shang , J. Z. Zhao , D. J. Gawryluk , M. Shi , M. Medarde , E. Pomjakushina , T. Shiroka , Phys. Rev. B 2020, 101, 214518.

[31] D. Y. Yan , M. Yang , C. X. Wang , P. B. Song , C. J. Yi , Y. G. Shi , Supercond. Sci. Tech. 2021, 34, 035025.

[32] Z. Cui , Y. Qian , W. Zhang , H. Weng , Z. Fang , Chinese Phys. Lett. 2020, 37, 087103.

[33] A. Huang , A. D. Smith , M. Schwinn , Q. Lu , T.‐R. Chang , W. Xie , H.‐T. Jeng , G. Bian , Phys. Rev. Mater. 2018, 2, 054205.

[34] D. Yan , D. Geng , Q. Gao , Z. Cui , C. Yi , Y. Feng , C. Song , H. Luo , M. Yang , M. Arita , S. Kumar , E. F. Schwier , K. Shimada , L. Zhao , K. Wu , H. Weng , L. Chen , X. J. Zhou , Z. Wang , Y. Shi , B. Feng , Phys. Rev. B 2020, 102, 205117.

[35] J.‐W. Yeh , S.‐K. Chen , S.‐J. Lin , J.‐Y. Gan , T.‐S. Chin , T.‐T. Shun , C.‐H. Tsau , S.‐Y. Chang , Adv. Eng. Mater. 2004, 6, 299.

[36] S. Akrami , P. Edalati , M. Fuji , K. Edalati , Mater. Sci. Eng. R: Rep. 2021, 146, 100644.

[37] H. Xiang , Y. Xing , F.‐Z. Dai , H. Wang , L. Su , L. Miao , G. Zhang , Y. Wang , X. Qi , L. Yao , H. Wang , B. Zhao , J. Li , Y. Zhou , J. Adv. Ceram. 2021, 10, 385.

[38] S.‐C. Luo , W.‐M. Guo , K. Plucknett , H.‐T. Lin , J. Am. Ceram. Soc. 2022, 11, 805.

[39] X. Yan , L. Constantin , Y. Lu , J.‐F. Silvain , M. Nastasi , B. Cui , J. Am. Ceram. Soc. 2018, 101, 4486.

[40] P. Sarker , T. Harrington , C. Toher , C. Oses , M. Samiee , J.‐P. Maria , D. W. Brenner , K. S. Vecchio , S. Curtarolo , Nat. Commun. 2018, 9, 4980. 30478375 10.1038/s41467-018-07160-7 PMC6255778

[41] F. Von Rohr , M. J. Winiarski , J. Tao , T. Klimczuk , R. J. Cava , Proc. Natl. Acad. Sci. USA 2016, 113, E7144. 27803330 10.1073/pnas.1615926113 PMC5135312

[42] P. Kozelj , S. Vrtnik , A. Jelen , S. Jazbec , Z. Jaglicic , S. Maiti , M. Feuerbacher , W. Steurer , J. Dolinsek , Phys. Rev. Lett. 2014, 113, 107001. 25238377 10.1103/PhysRevLett.113.107001

[43] R. Prozorov , V. G. Kogan , Phys. Rev. Appl. 2018, 10, 014030.

[44] L. Zeng , Z. Wang , J. Song , G. Lin , R. Guo , S.‐C. Luo , S. Guo , K. Li , P. Yu , C. Zhang , W.‐M. Guo , J. Ma , Y. Hou , H. Luo , Adv. Funct. Mater. 2023, 33, 2301929.

[45] H. Shu , W. Zhong , J. Feng , H. Zhao , F. Hong , B. Yue , (Preprint) arXiv:2307.16438, v1, submitted: Jul 2023.

[46] L. Zeng , X. Hu , S. Guo , G. Lin , J. Song , K. Li , Y. He , Y. Huang , C. Zhang , P. Yu , J. Ma , D.‐X. Yao , H. Luo , Phys. Rev. B 2022, 106, 134501.

[47] L. Zeng , X. Hu , N. Wang , J. Sun , P. Yang , M. Boubeche , S. Luo , Y. He , J. Cheng , D.‐X. Yao , H. Luo , J. Phys. Chem. Lett. 2022, 13, 2442. 35263107 10.1021/acs.jpclett.2c00404

[48] P. K. Jha , S. P. Sanyal , Phys. C 1996, 271, 6.

[49] P. K. Jha , S. P. Sanyal , Phys. C 1996, 261, 259.

[50] M. Talati , P. K. Jha , Phys. Rev. B 2006, 74, 134406.

[51] J. Guo , H. Wang , F. Von Rohr , Z. Wang , S. Cai , Y. Zhou , K. Yang , A. Li , S. Jiang , Q. Wu , R. J. Cava , L. Sun , Proc. Natl. Acad. Sci. USA 2017, 114, 13144. 29183981 10.1073/pnas.1716981114 PMC5740615

[52] S.‐C. Luo , W.‐M. Guo , Z.‐L. Fang , K. Plucknett , H.‐T. Lin , J. Eur. Ceram. Soc. 2022, 42, 336.

[53] H. K. Mao , J. Xu , P. M. Bell , J. Geophys. Res. 1986, 91, 4673.

[54] A. Van De Walle , Calphad 2009, 33, 266.

[55] G. Kresse , J. Hafner , Phys. Rev. B 1993, 47, 558. 10.1103/physrevb.47.558 10004490

[56] G. Kresse , J. Furthmüller , Phys. Rev. B 1996, 54, 11169. 10.1103/physrevb.54.11169 9984901

[57] J. P. Perdew , K. Burke , M. Ernzerhof , Phys. Rev. Lett. 1997, 78, 1396. 10.1103/PhysRevLett.77.3865 10062328

[58] P. E. Blöchl , Phys. Rev. B 1994, 50, 17953. 10.1103/physrevb.50.17953 9976227

