# Supplementary material for: Superconductivity in the High‐Entropy Ceramics Ti0.2Zr0.2Nb0.2Mo0.2Ta0.2C x with Possible Nontrivial Band Topology
Source: Adv Sci (Weinh). 2023 Dec 5;11(5):2305054. doi: 10.1002/advs.202305054 (PMC10837384; doi:10.1002/advs.202305054)
Supplement: Supplementary file 1 — Supporting Information [file ADVS-11-2305054-s001.pdf]

ADVANCED SCIENCE
Open Access

Supporting Information

for *Adv. Sci.*, DOI 10.1002/advs.202305054

Superconductivity in the High-Entropy Ceramics $Ti_{0.2}Zr_{0.2}Nb_{0.2}Mo_{0.2}Ta_{0.2}C_x$ with Possible Nontrivial Band Topology

Lingyong Zeng, Xunwu Hu, Yazhou Zhou, Mebrouka Boubeche, Ruixin Guo, Yang Liu, Si-Chun Luo, Shu Guo, Kuan Li, Peifeng Yu, Chao Zhang, Wei-Ming Guo, Liling Sun, Dao-Xin Yao* and Huixia Luo**

Supporting Information

Superconductivity in the high-entropy ceramics $Ti_{0.2}Zr_{0.2}Nb_{0.2}Mo_{0.2}Ta_{0.2}C_x$ with possible nontrivial band topology

Lingyong Zeng[1,#], Xunwu Hu[2,#], Yazhou Zhou[7,#], Mebrouka Boubeche[3], Ruixin Guo[4,5], Yang Liu[6], Si-Chun Luo[6], Shu Guo[4,5], Kuan Li[1], Peifeng Yu[1], Chao Zhang[1], Wei-Ming Guo[6], Liling Sun[7,], Dao-Xin Yao[2,5*], Huixia Luo[1,*]*

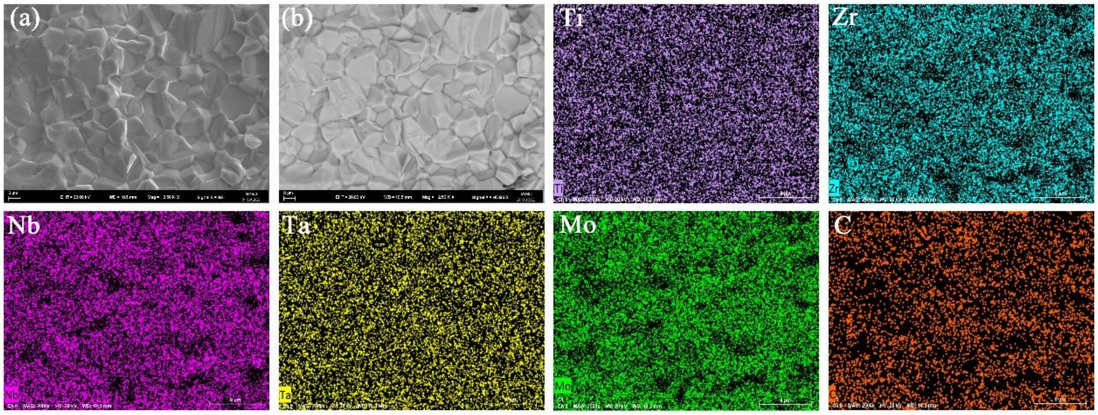

Figure S1 (a) SEM, (b) BSEM images, and EDX mappings of $Ti_{0.2}Zr_{0.2}Nb_{0.2}Mo_{0.2}Ta_{0.2}C$.

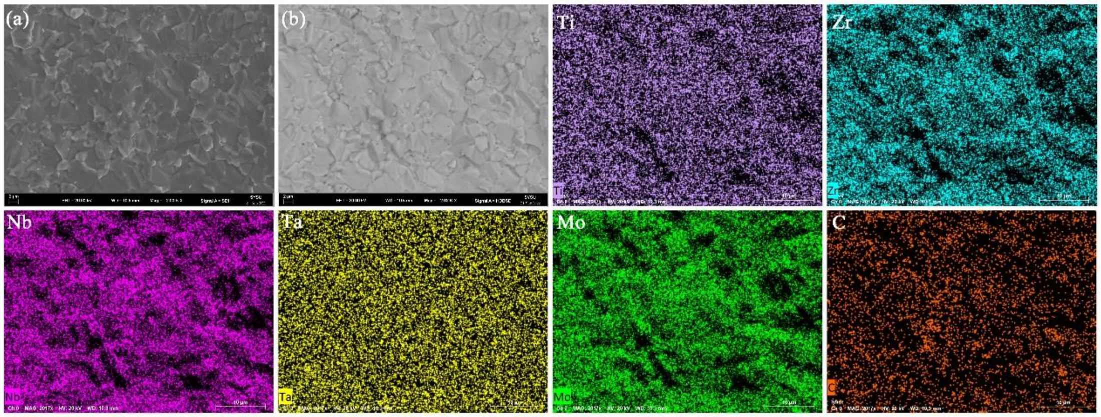

Figure S2 (a) SEM, (b) BSEM images, and EDX mappings of $Ti_{0.2}Zr_{0.2}Nb_{0.2}Mo_{0.2}Ta_{0.2}C_{0.8}$.

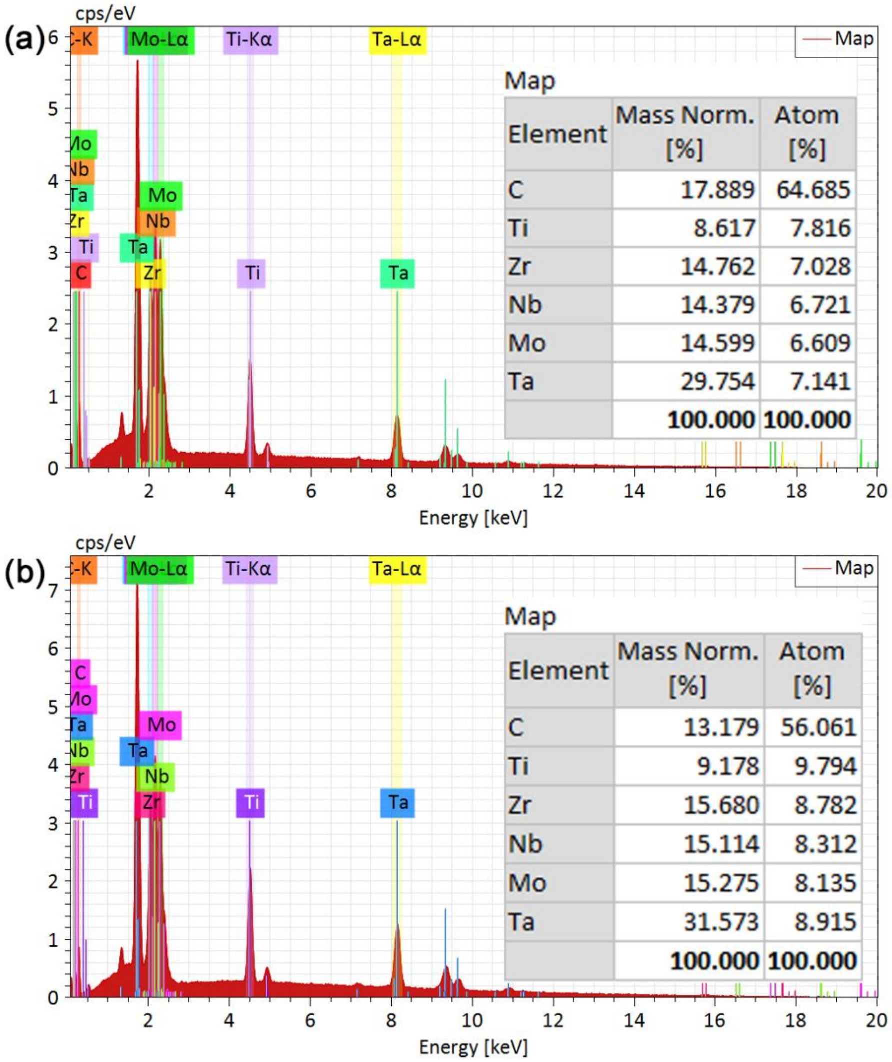

Figure S3 EDX spectrum of (a) $Ti_{0.2}Zr_{0.2}Nb_{0.2}Mo_{0.2}Ta_{0.2}C$ and (b) $Ti_{0.2}Zr_{0.2}Nb_{0.2}Mo_{0.2}Ta_{0.2}C_{0.8}$.

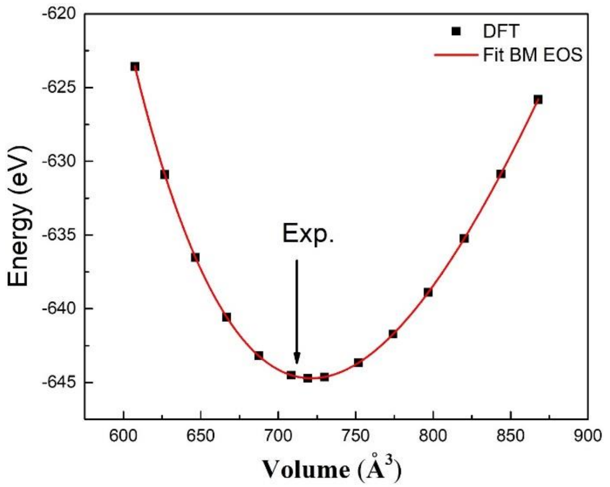

Figure S4 The calculated total energy as a function of volume.

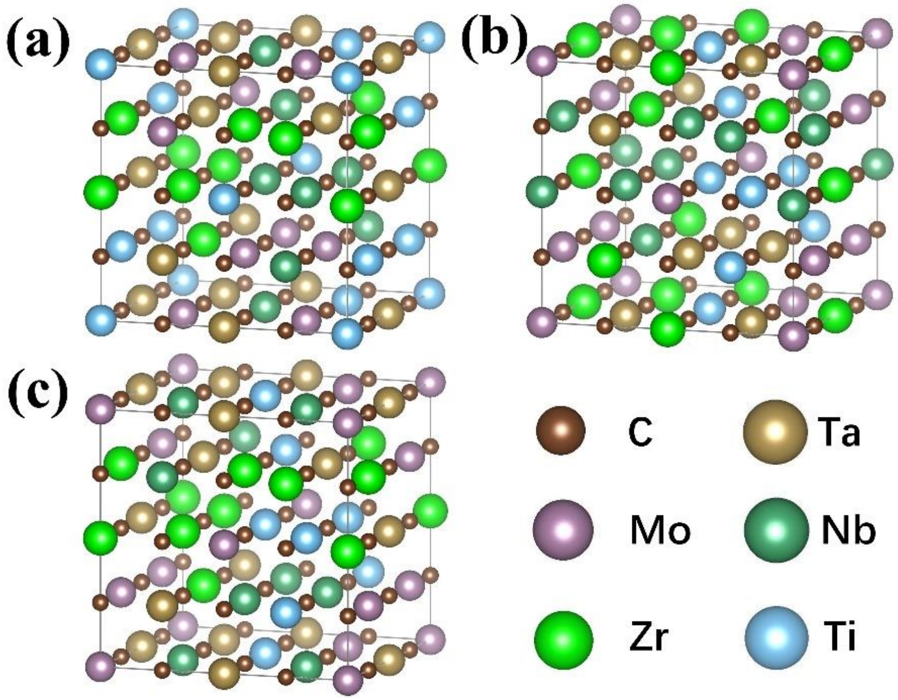

Figure S5 (a)-(c) Crystal structures of the three representative structures for Fig. 7(a)-(c).

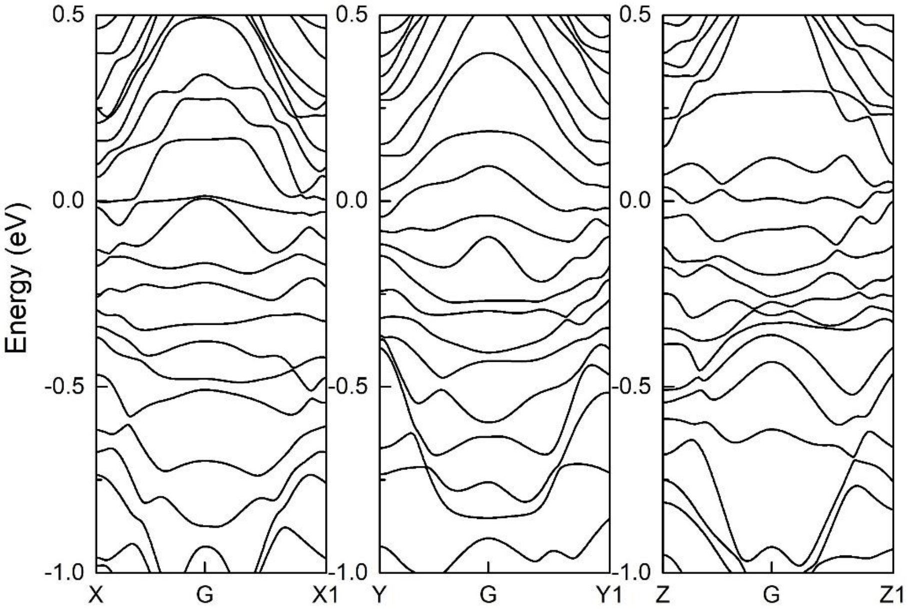

Figure S6 Electronic band structures of $Ti_{0.2}Zr_{0.2}Nb_{0.2}Mo_{0.2}Ta_{0.2}C$ (x = 0.8).

Table S1 The superconducting parameters of HECCs.

Property	$Ti_{0.2}Zr_{0.2}Nb_{0.2}Mo_{0.2}Ta_{0.2}C$	$Ti_{0.2}Zr_{0.2}Nb_{0.2}Mo_{0.2}Ta_{0.2}C_{0.8}$	$Ti_{0.2}Zr_{0.2}Nb_{0.2}Hf_{0.2}Ta_{0.2}C$[43]	$Mo_{0.2}Nb_{0.2}Ta_{0.2}V_{0.2}W_{0.2}C_{0.9}$[44]	$Ta_{0.25}Ti_{0.25}Nb_{0.25}Zr_{0.25}C$[44]
T_c (K)	4.00	2.65	2.35	3.40	5.70
μ_0H_{c1} (mT)	28.9(6)	10.0(2)	26.1		
μ_0H_{c2} (T)	3.2(6)	2.3(4)	0.5(1)	3.4	1.5
μ_0H^P (T)	7.40	4.90	4.35	6.29	10.55
$\xi_{GL}(0)$	100.5(3)	118.6(5)	261.8(4)		

(Å)			
$\lambda_{GL}(0)$ (Å)	1186	2192	-
Θ_D (K)	715	647	724
λ_{ep}	0.49	0.46	0.54
